# pH Sensitive Drug Delivery Behavior of Palmyra Palm Kernel Hydrogel of Chemotherapeutic Agent

**DOI:** 10.3390/gels9010038

**Published:** 2023-01-02

**Authors:** Kummara-Madhusudana Rao, Kummari Subba Venkata Krishna Rao, Ramasubba-Reddy Palem, Uluvangada-Thammaiah Uthappa, Chang-Sik Ha, Sung-Soo Han

**Affiliations:** 1 School of Chemical Engineering, Yeungnam University, 280 Daehak-Ro, Gyeongsan 38541, Republic of Korea; 2 Research Institute of Cell Culture, Yeungnam University, 280 Daehak-Ro, Gyeongsan 38541, Republic of Korea; 3 Department of Chemistry, Yogi Vemana University, Kadapa 516 003, India; 4 Department of Medical Biotechnology, Dongguk University, 32 Dongguk-ro, Ilsandong-gu, Goyang 10326, Republic of Korea; 5 Department of Polymer Science and Engineering, School of Chemical Engineering, Pusan National University, Busan 46241, Republic of Korea

**Keywords:** palmyra palm kernel, polysaccharide, hydrogels, pH-sensitive, 5-fluorouracil, colon cancer

## Abstract

This study examined the gel behavior of naturally-occurring palmyra palm kernel (PPK). Due to the presence of polysaccharide in PPK hydrogels, they exhibit excellent swelling behavior in response to pH. Chemotherapeutic drug 5-fluorouracil (5-FU) was encapsulated in these gels using an equilibrium swelling technique. It was found that 5-FU had an encapsulation efficiency of up to 62%. To demonstrate the drug stability in the gels, the PPK hydrogels were characterized using fourier transform infrared spectroscopy, differential scanning calorimetry, and X-ray diffraction. The results showed that the PPK hydrogel matrix contained molecularly dispersed 5-FU drug. The PPK hydrogel exhibited a denser structure and a rough surface, according to images obtained by scanning electron microscopy. In vitro release tests were carried out at pH 1.2 (gastric fluid) and 7.4 (intestinal fluid). The efficacy of the encapsulation and the release patterns were influenced by the network topology of the PPK hydrogel. The release patterns showed that 5-FU was released gradually over a time internal of more than 12 h. The findings suggest that naturally-occurring PPK hydrogels loaded with chemotherapeutic drugs could be employed to treat colon cancer.

## 1. Introduction

Polysaccharides from plant resources have a history of being included in dose formulations for effective drug administration. Significant potential has been shown for polysaccharides such as alginate, carrageenan, guar gum, gum arabic, and konjac glucomannan as drug delivery methods for controlled release [1]. Therefore, drug delivery carriers such as microparticles, beads, tablets, and crosslinked hydrogels composed of natural materials are important for drug delivery applications. Among these, hydrogels are very important because of their responsiveness, degradability, and stability [2,3].

Recently, several hydrogels were developed from a combination of natural-natural and natural-synthetic polymers. In this study, the gel behavior of palmyra palm kernel was identified. Palmyra palms are economically useful and cultivated widely in tropical regions. The plant has been used traditionally as a stimulant, anti-leprotic, diuretic, and antiphlogistic. The palmyra palm is one of the most important trees in India, and has some 800 different uses. Indian (Andhra Pradesh) fruit, known as toddy palm seeds (Taati munjalu), is a delicate halwa/jelly-like fresh fruit beloved for its sweet, soft flesh and reviving sugary water inside. The kernel has a moisture content of 92.6%, 0.46% protein, 1% fat, and 6.29% carbs [4]. The kernel has mostly polysaccharides such as pectin, pullulan, and mannose-cellulose [5,6,7]. However, complete swelling properties, such as pH swelling, are not reported.

Antineoplastic agent 5-FU is an acidic, water-soluble, hydrophilic medication frequently used in clinical chemotherapy to treat solid tumors. With the antimetabolite 5-fluorouracil (5-FU), a common component of cancer chemotherapy, it is possible to avoid scarring after trabeculectomy and increase the likelihood of successful long-term retinal reattachment [8,9]. The intravenously-administered 5-FU in the treatment of colon cancer results in severe systemic effects, due to its toxic effects on the normal cells in the body [10]. The primary mechanism of action of the medication is as a thymidylate synthase inhibitor, which prevents DNA synthesis, interferes with nucleic acid synthesis, and eventually stops cell proliferation. pH-sensitive hydrogels are crucial for colon cancer drug delivery using 5-FU as model chemotherapeutic agent. Over the last decade, several pH-sensitive carriers, such as nanoparticles, microspheres, hydrogels, and enteric-coated materials, have been used for colon cancer drug delivery [11,12,13,14,15,16]. 

In the present contribution, 5-FU is encapsulated in palmyra palm kernel (PPK) hydrogels. PPK hydrogels can potentially be applied for colon cancer drug delivery based on their swelling and pH-dependent release behavior. This study examined their release characteristics. To the best of the authors’ knowledge, PPK was identified as a hydrogel for the first time because it could swell in aqueous media and show similar properties to chemically-synthesized hydrogels.

## 2. Results and Discussion

### 2.1. Development of 5-FU Loaded PPK Hydrogels and Its Characterization

**Figure 1:**
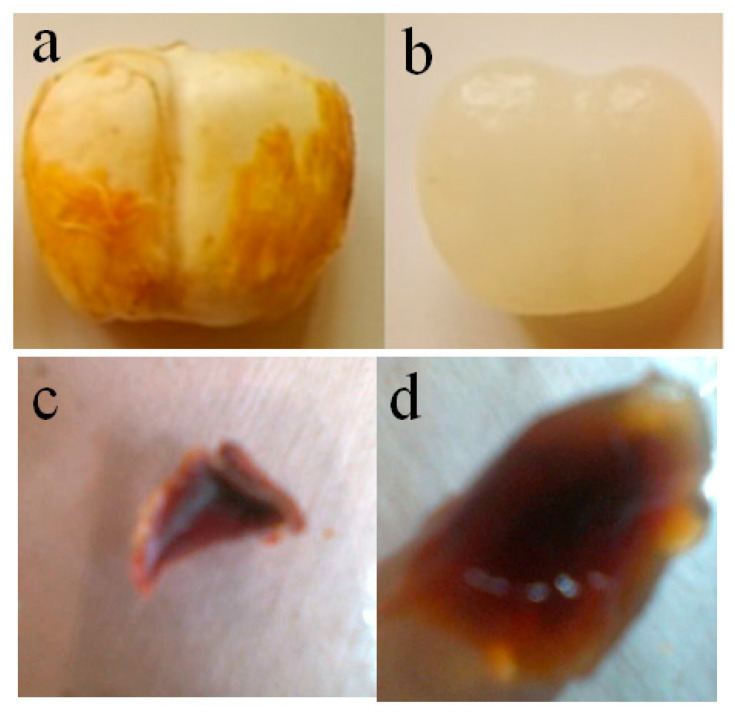
Digital photographs of (**a**) PPK fruit, (**b**) PPK after seed removal, (**c**) dry PPK hydrogel, and (**d**) swollen PPK hydrogel (photographs were captured using Nikon-D5200, Japan Optical Industries Co., Ltd.).

For drug delivery applications, many researchers have developed hydrogels composed of natural carbohydrate polymers, such as chitosan, sodium alginate, pectin, carrageenan, and dextrin. The present study developed naturally-occurring palmyra palm fruit hydrogels (Figure 1); the PPK hydrogels have many hydrophilic units and exhibit pH-sensitive properties. The presence of polysaccharides and a number of hydrophilic functionalities in the hydrogels was thought to be the cause of the pH sensitivity. Assoi et al. developed a method for the extraction of pectin polysaccharide from repined and young sugar palms through microwave-assisted extraction [5]. The PPK hydrogels are safe and economically useful. The pH sensitivity is also expected because pectin polysaccharide is present in palm fruit. Therefore, 5-FU was successfully encapsulated in the PPK hydrogels by immersing PPK hydrogel in a known concentration of 5-FU solution. The encapsulation efficiencies were also calculated, and Table 1 lists their values. The 5-FU-PPK hydrogels could be beneficial for drug delivery in the treatment of colon cancer.

**Figure 2:**
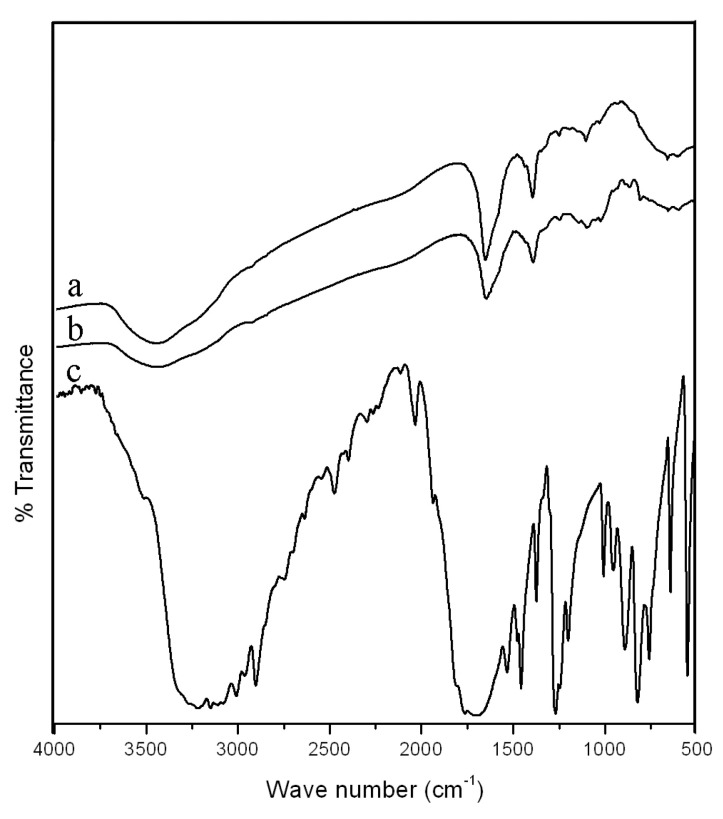
FTIR spectra of PPK hydrogel for P2 formulation: (a) pure PPK hydrogel, (b) 5-FU-PPK hydrogel, and (c) 5-FU.

FTIR, DSC, XRD, and SEM were used to characterize the PPK hydrogels. These characterization techniques provided useful information on PPK hydrogels. Figure 2 shows the FT-IR spectra of (a) PPK hydrogel (P2), (b) 5-FU-PPK hydrogel (P2), and (c) 5-FU. The sharp band at 2924 cm^−1^ indicated C-H stretching, whereas the broadband at 3436 cm^−1^ was linked to OH stretching. Additional distinguishing signals were identified for the C-O-H bending at 1451 cm^−1^, C-O-C stretching at 1107 cm^−1^ and C-O stretching vibrations at 1037 cm^−1^. The carboxylic C=O group was given the band at 1656 cm^−1^. On the other hand, a peak at 1656 cm^−1^ (carboxylic C=O) was shifted to lower frequency (1648 cm^−1^) for 5-FU-PPK hydrogel, suggesting hydrogen bonding interactions between PPK functional groups and 5-FU.

**Figure 3:**
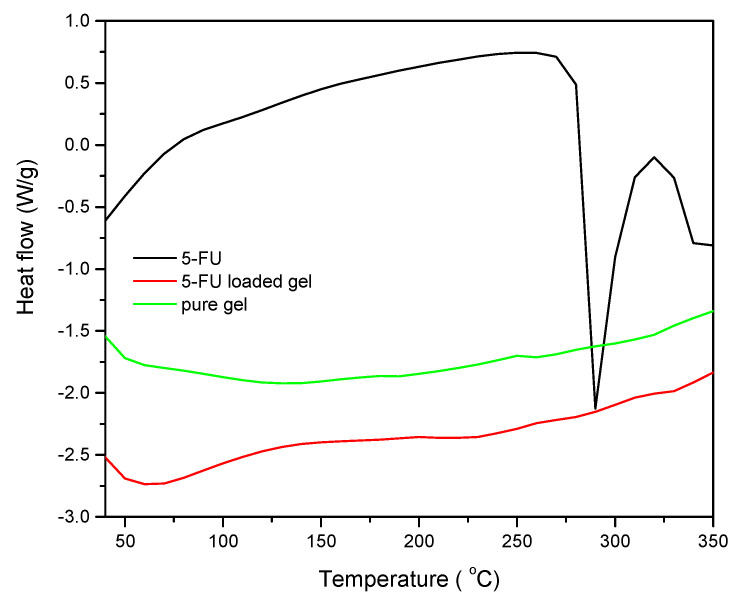
DSC curves of PPK hydrogel for P2 formulation: pure PPK hydrogel, 5-FU-PPK hydrogel, and 5-FU.

Studies using DSC and XRD are crucial for determining the drugs’ characteristics after being encapsulated in hydrogels. Figure 3 displays the DSC values for PPK hydrogel (P2), 5-FU-PPK hydrogel (P2), and 5-FU. Due to melting and polymorphism of 5-FU, the DSC curve showed a strong peak at 285 °C (Curve C) [15,16]. On the other hand, assuming that the drug is dispersed molecularly in the PPK gel, this peak did not show up in the case of the 5-FU-PPK hydrogels.

**Figure 4:**
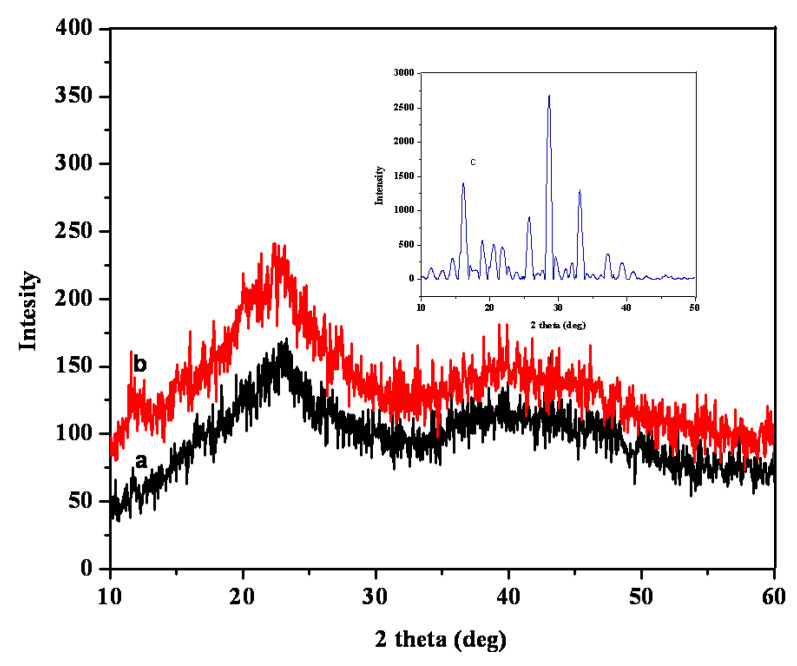
XRD patterns of PPK hydrogel for P2 formulation: (a) pure PPK hydrogel, (b) 5-FU-PPK hydrogel, and (c) 5-FU.

**Figure 5:**
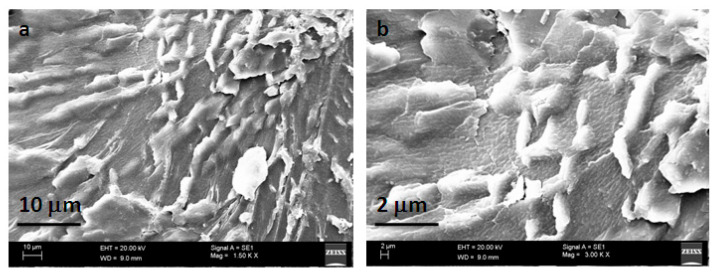
SEM images of PPK hydrogel for P2 formulation: (**a**) low magnification, and (**b**) high magnification ([**a**] ×1.50KX; [**b**] ×3.00KX).

Figure 4 shows the XRD patterns of pure 5-FU, 5-FU-PPK hydrogel (P2), and PPK hydrogel (P2). Due to the drug’s crystalline structure, pure 5-FU had peaks at 17 °C, 29 °C, and 32 °C. However, in the 5-FU loaded PPK hydrogels, the crystalline peaks of 5-FU vanished, indicating that the 5-FU molecules were dispersed throughout the hydrogel matrix [17,18]. Due to the hydrogels’ amorphous structures, both drug-loaded and pure hydrogels showed peaks at 23 °C and 41.2 °C. SEM pictures of the PPK hydrogels for the P2 formulation are shown in Figure 5. The surface morphology of pure PPK hydrogel revealed a denser structure and a rough surface. 

### 2.2. Swelling Study and pH-Responsive Property of PPK Hydrogels

**Figure 6:**
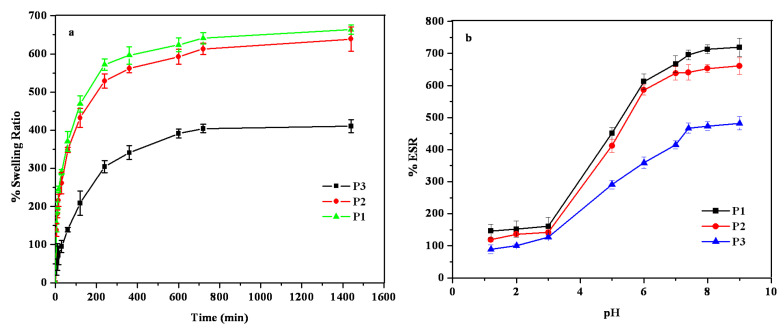
(**a**) Swelling studies of PPK hydrogels in DDW at 37 °C and (**b**) %ESR of PPK hydrogels at various pH conditions.

PPK hydrogels were permitted to swell in DDW and various pH solutions (Figure 6). The swelling of the three types of PPK hydrogels depends on their aging time. Figure 6a depicts the PPK hydrogels’ swelling behavior in DDW as a function of time. The swelling order of the PPK hydrogels was P1 > P2 > P3. The pH swelling of all hydrogels was examined at different pH solutions. The %ESR values of PPK hydrogels in the pH solutions depends on the functional groups in the hydrogels. Figure 6b displays the swelling ratio of PPK hydrogel (P2) in various pH solutions. The swelling ratios for all hydrogels increased slowly up to pH 5.0 and significantly increased at higher pH. It is expected that PPK hydrogels have carboxylic functional groups, which are responsible for its ionization under different pH conditions. At lower pH, the carboxylic groups were converted to –COOH, and hydrogen bonding developed. A higher pH, on the other hand, caused the carboxylic groups to become ionic and the repelling force to become stronger, which promoted swelling. Similar to chemically-synthesized hydrogels, the newly-identified natural hydrogels exhibited excellent swelling behavior in aqueous solutions at various pH.

### 2.3. In Vitro Release Study

**Figure 7:**
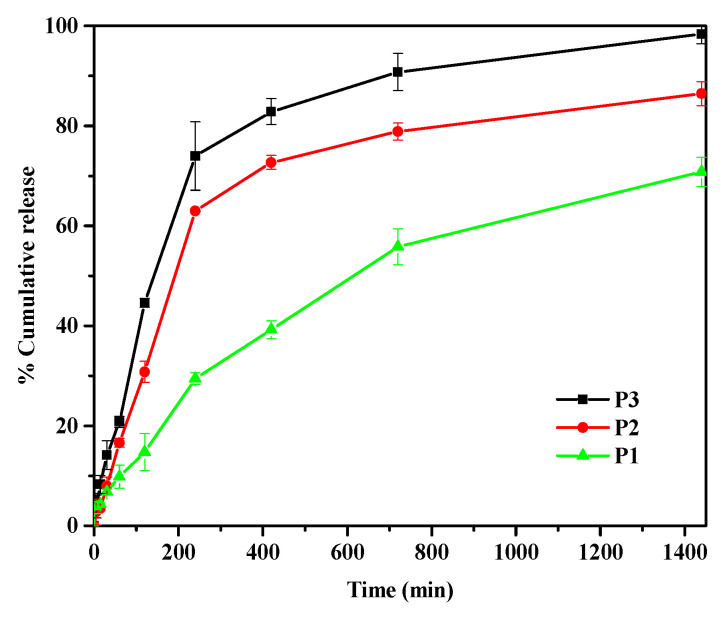
In vitro release behavior of 5-FU through PPK hydrogels in pH 7.4 at 37 °C for P1, P2, and P3.

The in vitro release performance of different PPK hydrogels (P1, P2, P3) loaded with 5-FU drug was analyzed in pH 7.4 conditions at 37 °C. As can be seen in Figure 7, the network structure of PPK hydrogel produced from PPK fruits with various aging periods affects the percentage of 5-FU release from PPK hydrogel. As the aging time increased, the PPK hydrogels showed a more rigid structure. The order of the 5-FU release trend from the PPK hydrogels was P1 > P2 > P3. The rigid network structure with microvoid contracting was responsible for the lowest release of 5-FU from the P3 formulation. Therefore, the buffer molecules could not travel easily into the networks of the PPK hydrogels. In general, the release profile of drugs from hydrogels depends on the crosslinking between polymer chains. At a higher level of crosslinking, the drug molecules cannot easily be diffused out from the hydrogel, due to rigid network structure [17]. Similarly, the release of 5-FU sped up from the softer PPK hydrogels, while becoming slower from the harder PPK hydrogels.

**Figure 8:**
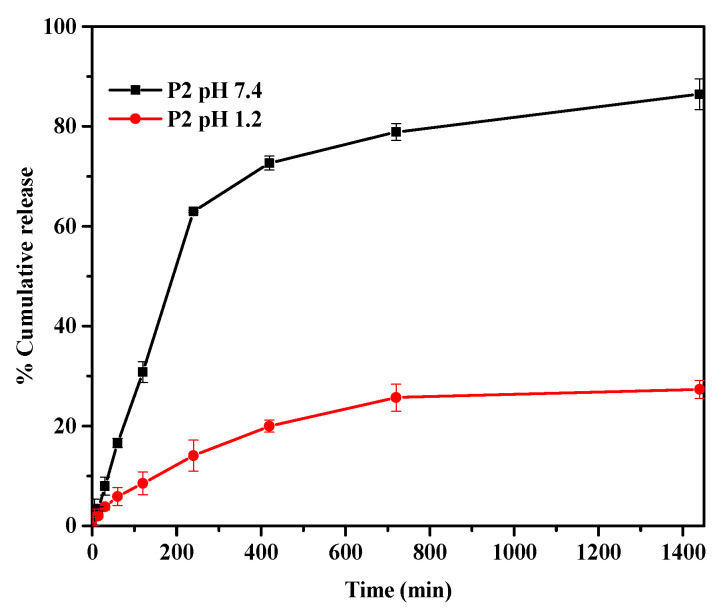
In vitro release behavior of the PPK hydrogel (P2) in pH 1.2 and 7.4 buffer media at 37 °C.

The in vitro release of 5-FU from PPK hydrogels (P2 formulation) were carried out in gastro-intestinal fluids at 37 °C (Figure 8). The release patterns were found to depend on the pH condition. The % of 5-FU was higher at higher pH conditions, whereas the %5FU was lower for gastric fluid (pH 1.2). This finding can be explained on the basis of functional groups that exist in the PPK hydrogels. PPK contains pectin-based polysaccharides, which have ionizable carboxylic acid groups that can influence drug release. Negative charge repulsion forces were generated at higher pH conditions (7.4) because of the ionization of carboxylic groups, thereby resulting in maximum drug release. At pH 1.2, the ionization repulsive forces were decreased due to the protonation of carboxylate ions, which meant drug release was slower. Thus, 5-FU release was dependent on the pH-responsive behavior of PPK hydrogel, which can have benefits for drug delivery in colon cancer patients. Moreover, the release profiles were fitted using Korsmeyer-Peppas equation to corroborate the release mechanism as follows [19]:

$$\frac{M_{t}}{M_{\infty }}A=k_{p}t^{n}$$

where k_p_ is the rate constant, n is the release exponent, and M_t_/M_∞_ is the percentage of the drug released at time t. The release mechanism was evaluated using the estimated n value.

For all formulations, n and k were calculated using the least square method, and the results are shown in Table 1 as a summary. According to the equation, the log value of the percentage of 5-FU release from PPK hydrogel was plotted as a function of log time. Fickian diffusion governs the drug release from the polymer matrix if n < 0.5. For n > 0.5, anomalous or non-Fickian drug diffusion occurs. Release kinetics that are completely non-Fickian or case II type for n = 1. The n values of the current data were 0.26 to 0.35, indicating a Fickian diffusion pattern. Thus, the n values are affected by the network topology of PPK hydrogel.

## 3. Conclusions

The pH swelling responsive behavior of palmyra fruit hydrogels collected from palmyra palm tree was reported. It is interesting to note that in alkaline conditions, the PPK hydrogels showed greater swelling behavior. When 5-FU was introduced to the PPK hydrogels as a model drug, its encapsulation efficiency reached 62%. DSC and XRD revealed the molecular dispersion of 5-FU in the PPK hydrogels. In vitro release tests showed that PPK hydrogel released 5-FU in intestinal fluid more quickly than in stomach fluid. The naturally-occurring PPK hydrogels were stable for more than one year. The economically cheap and safe natural hydrogels were found to be an efficient carrier for colon cancer drug delivery of 5-FU.

## 4. Materials and Methods

Palmyra palm fruits were collected from the surroundings of the Yaganti temple, Kurnool district, Andhrapradesh, India. The fruit kernel seed was removed (Figure 1a,b show digital photographs of the palmyra palm kernel before and after removing the seed, respectively), and the white jelly kernel was then cut into small pieces and immersed repeatedly in water and aqueous methanol for three days to remove any water-soluble sugars and other materials. The resulting pieces were air-dried and then dried at 40 °C in an electronically-controlled hot air oven. The pieces were used further for swelling, drug loading, and characterization studies. In this study, three types of fruits were used according to their aging time (soft, hard, and harder fruits, named P1, P2, and P3 and listed in Table 1). Because many hydrophilic units are present in the palmyra palm fruit, they are swellable in water. Figure 1c,d present digital photographs of dry PPK gel and swollen PPK gel, respectively. 5-Fluorouracil (5-FU) was purchased from Himedia, India.

### 4.1. Swelling Studies

Mass measurements were used to determine the swelling capacity of PPK hydrogels when placed in DDW at 37 °C. First, the dry PPK hydrogels were accurately weighed and submerged in DDW for various time periods. After swelling, the PPK hydrogels were carefully removed and wiped to remove any surface-adhered water. The pH-sensitive properties of PTK hydrogel were measured by recording the swelling of the hydrogels at different pH levels (pH 1.2, 2.0, 3.0, 5.0, 6.0, 7.0, 7.4, 8.0, and 9.0). The percentage swelling (%SR) ratio and the equilibrium swelling (%ESR) ratio were calculated in accordance with the formulae below:

$$\%\;\mathrm{SR}=\frac{W_{s}-W_{d}}{W_{d}}\times 100$$

$$\%\;\mathrm{ESR}=\frac{W_{e}-W_{d}}{W_{d}}\times 100$$

where W_s_ is the weight of the gel while it is swelling at time t, W_d_ is the weight of the hydrogel when it is dry, and W_e_ is the weight of the gel after it has reached equilibrium in the water. 

### 4.2. 5-FU Loading 

5-FU was loaded into PPK hydrogels by an equilibrium swelling method in drug solutions according to previously reported work [17]. The hydrogels were then allowed to swell for 24 h at 37 °C in a known amount of drug solution. 5-FU only dissolves in water at a very low concentration (13 mg/mL). On the other hand, its sodium salt is 65 mg/mL solubilized. Gel discs were submerged in a 5-FU aqueous solution, which was neutralized with NaOH, to load the maximal amount of 5-FU into the PPK hydrogel network. The 5-FU in the solvent was adsorbed onto the hydrogels during this process.

5-FU loading efficiency of 5FU-PPK hydrogels was assessed spectrophotometrically. To extract the 5-FU from the PPK hydrogels, a 5-FU loaded PPK hydrogel disc was submerged in 20 mL of buffer solution and rapidly agitated for 48 h. After filtering, the solution was measured with a UV-Vis spectrophotometer at a wavelength of 270 nm. The following equations were utilized for the calculation of drug loading (%DL) and encapsulation efficiency (%EE):

$$\%\;\mathrm{Drug}\;\mathrm{loading}=\frac{\mathrm{Amount}\;\mathrm{of}\;\mathrm{drug}\;\mathrm{in}\;\mathrm{hydrogel}}{\mathrm{Amount}\;\mathrm{of}\;\mathrm{hydrogel}}\times 100$$

$$\%\;\mathrm{Encapsulation}\;\mathrm{Efficiency}=\frac{\mathrm{Actual}\;\mathrm{loading}}{\mathrm{Theoritical}\;\mathrm{loading}}\times 100$$

Table 1 lists the data for various formulations.

### 4.3. In Vitro 5-FU Release 

The release of 5-FU from PPK hydrogels was carried out employing dissolution experiments using a LabIndia DS-8000 tablet dissolution tester system fitted with eight baskets. The drug release performance was measured at 37 °C with rotation speed of 100 rpm at different time intervals (5, 15, 30, 60, 120, 140, 420, 720, and 1440 min). The 5-FU release from the 5-FU-PPK hydrogels was examined in fluids with gastric pH values of 1.2 and intestinal pH values of 7.4. Aliquot samples were taken out and examined using UV spectrophotometry (UV-3092, LAB INDIA, Mumbai, India) at a maximum wavelength of 270 nm at regular intervals.

### 4.4. Characterization

Fourier transform infrared spectroscopy (FTIR) was performed on the PPK hydrogels, drug-loaded PPK hydrogel, and pure 5-FU using a Perkin Elmer (Impact 410, Wisconsin, MI, USA) instrument scanning between 4000 and 500 cm^−1^. Before scanning spectra, under a hydraulic pressure of 600 dynes/m^2^, the hydrogels were finely powdered with KBr to make the pellets. The differential scanning calorimetry (DSC) curves of the PPK hydrogels, 5-FU-PPK hydrogel, and 5-FU were analyzed using a sequential thermal analyzer (TA instruments-Model-SDT-Q600). The test was performed at a heating rate of 10 °C/min with 100 mL/min purging rate of N2 environment. The PPK hydrogels were observed using scanning electron microscopy (SEM) (SUPRA25, Carl Zeiss AG, Jena, Germany) at two magnifications using acceleration voltage at 20 kV. 

## Figures and Tables

**Table 1:** In vitro release parameters and encapsulation efficiency values of various PPK hydrogels.

Formulation Code	n	r	Kp (×10^2^)	%EE
P1 (soft PPK)	0.3592	0.9686	0.2324	62.82
P2 (hard PPK)	0.2832	0.9867	0.8512	59.42
P3 (more harder PPK)	0.2691	0.9912	0.9862	54.69

## Data Availability

Not applicable.
